# The value of long noncoding RNAs for predicting the recurrence of endometriosis: A protocol for meta-analysis and bioinformatics analysis

**DOI:** 10.1097/MD.0000000000026036

**Published:** 2021-05-28

**Authors:** Yihong Chen, Xinghui Liu, Lei He

**Affiliations:** a Laboratory of the Key Perinatal Diseases, Key Laboratory of Birth Defects and Related Diseases of Women and Children, Ministry of Education; b Department of Obstetrics and Gynecology, West China Second University Hospital, Sichuan University, Chengdu, Sichuan Province, China.

**Keywords:** bioinformatics, endometriosis, long noncoding RNAs, meta-analysis, protocol, recurrence

## Abstract

**Background:**

As a gynecological disease, endometriosis (EM) seriously endangers the health of women at the age of childbearing and is closely related to long noncoding RNAs (lncRNAs). Current studies have discovered that there are differential expressions of many kinds of lncRNAs in EM. However, whether lncRNAs can be applied as a new marker for the prediction of the recurrence of EM is still controversial. In this study, meta-analysis and bioinformatics analysis were carried out to explore the value of lncRNAs as a predictor of the recurrence of EM and to analyze its biological role.

**Methods:**

PubMed, Embase, and Web of Science databases were searched through computer and the articles published from the self-built database to April 2021 were collected. According to the inclusion and exclusion criteria, the literature was screened, and the quality of the inclusion study was evaluated. Stata 16.0 software was used for meta-analysis. The co-expression genes related to lncRNAs were screened by online tool Co-LncRNA. Then David for Gene Ontology and Kyoto Encyclopedia of Genes and Genomes analysis were conducted. A competitive endogenous RNA network that may exist in lncRNAs through Starbase was built.

**Results:**

The results of this meta-analysis would be submitted to peer-reviewed journals for publication.

**Conclusion:**

This meta-analysis could provide high-quality evidence support for lncRNAs, so as to predict the recurrence of EM. At the same time, we use bioinformatics technology to predict and analyze its biological effects, which provides a theoretical basis for further experimental verification.

**Ethics and dissemination:**

The private information from individuals will not be published. This systematic review also should not damage participants’ rights. Ethical approval is not available. The results may be published in a peer-reviewed journal or disseminated in relevant conferences.

**OSF Registration Number:**

DOI 10.17605/OSF.IO/MF3QJ.

## 1 Introduction

Characterized by the appearance of endometrial matrix and glands outside the uterine cavity,^[1]^ endometriosis (EM) is a gynecological disease. The pathogenesis of EM is multifactorial, and hormone, immune, environmental and genetic factors are all related to it.^[2,3]^ Although the exact pathogenesis is still unclear, genetic factors, especially epigenetic factors, play an important role.^[4]^ After treatment, EM has a high recurrence rate, and no effective recurrence predictors could be found. To this end, finding noninvasive biomarkers for EM has been an ongoing and challenging problem.

As an important branch of epigenetic research, noncoding RNA can regulate the expression of related genes, then play a significant role in abnormal proliferation, apoptosis, invasion, steroid hormone metabolism and its receptor expression, epithelial-mesenchymal transformation, angiogenesis and other processes of endometrial cells, and promote the occurrence and development of EM.^[5]^ An increasing number of evidences indicate that long noncoding RNAs (lncRNAs) are involved in the genesis and development of EM in various ways.^[5–11]^ In addition, the potential of lncRNAs in improving the early diagnosis, evaluation, and treatment of EM has been widely explored.^[12]^

In recent years, related studies have proved that lncRNAs have important clinical significance in predicting the recurrence of EM.^[13–16]^ However, the results of each study are inconsistent. For this reason, this study performed a meta-analysis to evaluate the accuracy of lncRNAs in predicting the recurrence of EM, and to provide evidence support for the application of clinical noninvasive markers. In this study, in order to further understand the biological role of lncRNAs in EM, a competitive endogenous RNA network was constructed by means of bioinformatics to reveal the expression and function of regulatory genes, which provide a theoretical basis for further experimental verification.

## 2 Methods

### 2.1 Study registration

This meta-analysis protocol is based on the Preferred Reporting Items for Systematic Reviews and Meta-analysis Protocols (PRISMA-P) statement guidelines.^[17]^ The protocol of the systematic review was registered on Open Science Framework, and the registration number is DOI 10.17605/OSF.IO/MF3QJ.

### 2.2 Data sources and retrieval strategy

We searched the Web of Science, PubMed, and EMBASE databases to identify all potentially eligible articles from inception to April 2021. The detailed search strategies are listed in Table 1.

**Table 1:** Search strategy in PubMed database.

Number	Search terms
#1	RNA, Long Untranslated[MeSH]
#2	LINC RNA[Title/Abstract]
#3	LincRNAs[Title/Abstract]
#4	Long Intergenic Non-Protein Coding RNA[Title/Abstract]
#5	Long Non-Coding RNA[Title/Abstract]
#6	Long Non-Protein-Coding RNA[Title/Abstract]
#7	Long Noncoding RNA[Title/Abstract]
#8	Long ncRNA[Title/Abstract]
#9	Long ncRNAs[Title/Abstract]
#10	RNA, Long Non-Translated[Title/Abstract]
#11	Long Intergenic Non Protein Coding RNA[Title/Abstract]
#12	Long Non Coding RNA[Title/Abstract]
#13	Long Non Protein Coding RNA[Title/Abstract]
#14	Long Non-Translated RNA[Title/Abstract]
#15	Long Untranslated RNA[Title/Abstract]
#16	Non-Coding RNA, Long[Title/Abstract]
#17	Non-Protein-Coding RNA, Long[Title/Abstract]
#18	Non-Translated RNA, Long[Title/Abstract]
#19	Noncoding RNA, Long[Title/Abstract]
#20	RNA, Long Non Translated[Title/Abstract]
#21	RNA, Long Non-Coding[Title/Abstract]
#22	RNA, Long Non-Protein-Coding[Title/Abstract]
#23	RNA, Long Noncoding[Title/Abstract]
#24	Untranslated RNA, Long[Title/Abstract]
#25	ncRNA, Long[Title/Abstract]
#26	ncRNAs, Long[Title/Abstract]
#27	or/1–26
#28	Endometriosis[MeSH]
#29	Endometrioma[Title/Abstract]
#30	Endometriomas[Title/Abstract]
#31	Endometrioses[Title/Abstract]
#32	or/28–31
#33	Recurrence[MeSH]
#34	Diagnos^∗^[Title/Abstract]
#35	Sensitivity[Title/Abstract]
#36	Specificity[Title/Abstract]
#37	ROC curve[Title/Abstract]
#38	or/34–37
#39	#27 and #32 and #33 and #38

### 2.3 Inclusion criteria for study selection

#### 2.3.1 Inclusion criteria

1. The patients who were diagnosed with EM. At the same time, surgical treatment was performed.
2. The samples of lncRNA come from tissue, platelets, serum, peripheral blood mononuclear cells, plasma, and whole blood.
3. To explore the diagnostic value of lncRNAs on the diagnosis of recurrence of EM.
4. True positive, false positive, false negative, and true negative can be calculated based on the information in the literature.

#### 2.3.2 Exclusion criteria

1. Repeatedly published research.
2. Animal experiment.
3. Comments, case reports, conference summaries, and meta-analysis.
4. Insufficient data.

### 2.4 Data collection and analysis

**Figure 1:**
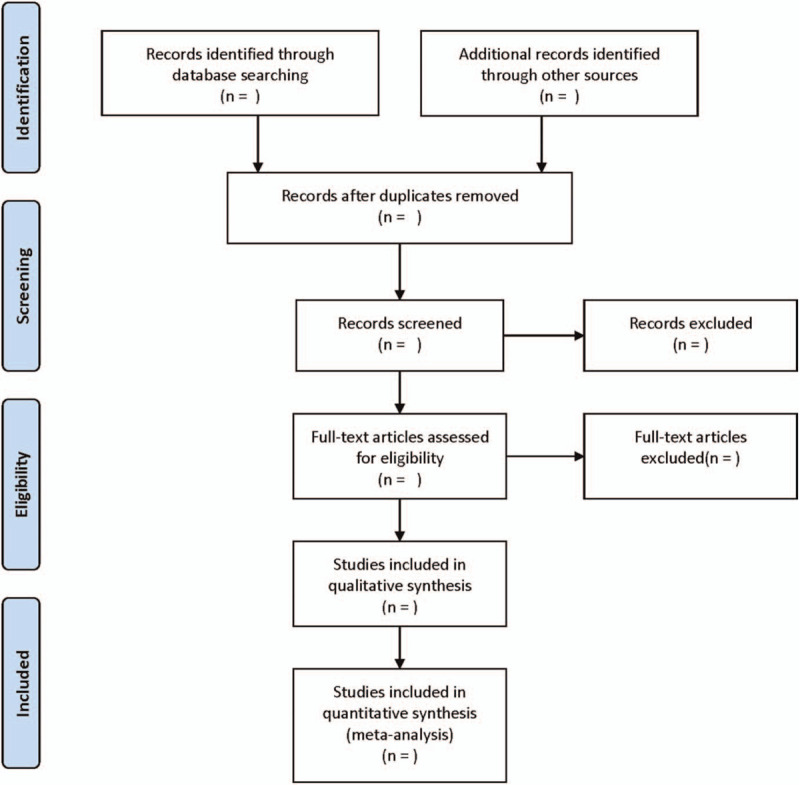
Flow diagram of study selection process.

The literature screening process is displayed in Figure 1. According to the unified inclusion and exclusion criteria, after the preliminary screening of the literature, the data of the included literature were extracted independently by 2 researchers and cross-checked to confirm whether the data were accurate or not. The data collected include the first author, the number of years published, the nationality of the study, the design of the study, the source of the sample, the detection method, the type of lncRNA, the longest follow-up period, outcome indicators, etc. Furthermore, in view of the fact that some studies only provide receiver operating characteristic curve, it is necessary to use Engauge Digitizer4.1 version to extract true positive, false positive, false negative, and true negative.^[18,19]^

### 2.5 Quality assessment

The risk of bias in the included studies was assessed using the Quality Assessment of Diagnostic Accuracy Studies 2 (QUADAS-2) score system.^[20]^

### 2.6 Outcome indicator

The outcomes include pooled sensitivity, specificity, positive likelihood ratio, negative likelihood ratio, diagnostic odds ratio, area under the curve, and their 95% confidence intervals.

### 2.7 Management of missing data

If there exists insufficient or missing data in the literature, we would only analyze the currently available data and discuss its potential value.

### 2.8 Statistical analysis

All statistical analyses were conducted using Stata 16.0 (STATA Corp, College Station, TX) software. We calculated the pooled sensitivity, specificity, positive likelihood ratio, negative likelihood ratio, diagnostic odds ratio, and their 95% confidence intervals. What is more, the pooled diagnostic value of lncRNAs through the summary receiver operated characteristic curve and area under the curve was tested. The threshold effects were detected by using Spearman correlation coefficient. The calculation of heterogeneity was caused by the nonthreshold effects of Cochrane-Q and I^2^ values, and a fixed-effect model (without obvious inhomogeneity) or a random-effects model (with significant heterogeneity) was employed to merge the data. The statistical test level was α = 0.05.

### 2.9 Additional analysis

#### 2.9.1 Subgroup analysis

According to the detection methods of lncRNAs, ethnicity, and the source of lncRNAs, we analyzed the subgroup.

#### 2.9.2 Sensitivity analysis

Sensitivity analysis was performed via sequential deletion of a single included study to test.

#### 2.9.3 Reporting bias

The publication bias was determined by carrying out Deeks funnel plot asymmetry test.

## 3 Bioinformatics analysis

### 3.1 Screening of genes related to the role of lncRNAs

The genes that may be related to the expression level of lncRNAs were screened by online tool Co-LncRNA (http://www.bio-bigdata.com/Co-LncRNA/), and the intensity of co-expression was expressed by interaction score.^[21]^

### 3.2 Enrichment analysis of Gene Ontology biological function and Kyoto Encyclopedia of Genes and Genomes signaling pathway

Gene Ontology and Kyoto Encyclopedia of Genes and Genomes analyses were performed on the screened co-expressed genes using the online tool David (https://david.ncifcrf.gov/).^[22]^

### 3.3 Construction of competitive endogenous RNA network of lncRNAs

The online tool starbase (http://starbase.sysu.edu.cn/index.php) was used to study the micro RNA molecules targeted by lncRNAs and downstream target mRNA molecules.

## 4 Ethics and dissemination

The content of this article does not involve moral approval or ethical review and would be presented in print or at relevant conferences.

## 5 Discussion

As an important member of the noncoding RNA family in vivo, lncRNAs play an important regulatory role in the occurrence and development of many diseases.^[23,24]^ In recent years, with the development of high-throughput sequencing technology, more and more attention has been paid to the important functions of lncRNAs. As a common disease in women at the age of childbearing, EM seriously affects their physiological health.^[25,26]^ Although the cause of the disease is not clear, genetic factors play an important role in it. Although scholars have revealed that a variety of differentially expressed lncRNAs may play an important role in the occurrence and development of EMs.^[27]^ Existing studies have indicated that lncRNAs promote the occurrence and development of EM through proliferation, invasion, metastasis, apoptosis, autophagy, and so on.^[9,28–30]^ Further screening of lncRNAs, with high specificity and sensitivity, has important clinical significance for the diagnosis, treatment, and prognosis of EM. In this study, meta-analysis and bioinformatics were used to screen lncRNA, with high specificity and sensitivity for predicting EM recurrence, thus constructing a competitive endogenous RNA network to reveal the occurrence and development of EM.

## Author contributions

**Conceptualization:** Lei He, Yihong Chen.

**Data curation:** Lei He, Yihong Chen.

Formal analysis: Xinghui Liu and Yihong Chen.

**Funding acquisition:** Lei He.

Methodology: Yihong Chen.

**Project administration:** Lei He.

**Software:** Xinghui Liu, Yihong Chen.

**Supervision:** Lei He.

**Validation:** Yihong Chen.

**Visualization:** Xinghui Liu, Yihong Chen.

**Writing – original draft:** Lei He, Yihong Chen.

**Writing – review & editing:** Lei He, Yihong Chen.
